# Histopathological Implications of *Aspergillus* Infection in Lung

**DOI:** 10.1155/2013/809798

**Published:** 2013-11-20

**Authors:** Naobumi Tochigi, Yoichiro Okubo, Tsunehiro Ando, Megumi Wakayama, Minoru Shinozaki, Kyoko Gocho, Yoshinobu Hata, Takao Ishiwatari, Tetsuo Nemoto, Kazutoshi Shibuya

**Affiliations:** ^1^Department of Surgical Pathology, Toho University School of Medicine, 6-11-1 Omori-Nishi, Ota-Ku, Tokyo 143-8541, Japan; ^2^Division of Pulmonary Medicine, Toho University School of Medicine, 6-11-1 Omori-Nishi, Ota-Ku, Tokyo 143-8541, Japan; ^3^Division of Chest Surgery, Toho University School of Medicine, 6-11-1 Omori-Nishi, Ota-Ku, Tokyo 143-8541, Japan; ^4^Department of Dermatology, Peking University First Hospital, 8# Xishiku Street, Xicheng District, Beijing 100034, China

## Abstract

This paper opens with a discussion on the significance of invasive fungal infections in advanced contemporary medicine, with an emphasis on the intractability of disease management and the difficulties of diagnosis. This is followed by a discussion concerning classification, histopathological features, and pathophysiology. While it has been largely accepted that *Aspergillus* species is recognized by cellular receptors and attacked by neutrophils, the radiological and macroscopic findings linking infection with neutropenia remain unconfirmed. In an effort to gain a better understanding of the pathophysiology and pathogenesis of invasive aspergillosis, we wish to emphasize the utility of radiological and histopathological examinations since these can provide detailed information on the extremely complex interaction between the causative microbes and tissue responses. A review of noninvasive or semi-invasive aspergillosis is also provided, with particular emphasis on chronic necrotizing pulmonary aspergillosis, which is recognized as a transition form of simple pulmonary aspergilloma and invasive pulmonary aspergillosis, although few findings have been reported in this area.

## 1. Introduction

Inflammation can be understood as the defense reaction of host against injury, which varies depending on the particular agent, tissue, and individual host characteristics [1–4]. Accordingly, infectious disease has been regarded as an inflammatory response caused by a microorganism that acts as an injury agent. Histopathological changes occurring at the site of infection and altered tissue structures generally result from an extensively complicated interaction between the causative microbes and tissue responses. Simplifying the issue to facilitate a better understanding of the processes involved, let us define infection as a pathognomonic condition induced by the interaction of pathogens with extracellular matrices. In the case of opportunistic infections, especially those involving invasive fungal infections, tissue responses against some pathogenic fungi are lowered, although the cause and extent of the defense mechanisms vary from case to case. In this regard, the features of lesions produced by an invasion of pathogenic fungi can be understood as a phenotypic representation resulting from an interaction between invasion by the causative fungi and variously lowered defense mechanisms of the host, as observed ubiquitously through a microscope.

It has generally been accepted that pulmonary aspergillosis can be divided into invasive pulmonary aspergillosis (IPA), chronic pulmonary aspergillosis (CPA), simple pulmonary aspergilloma (SPA), and allergic bronchopulmonary aspergillosis (ABPA) [5, 6]. Of these, ABPA can be regarded as being representative of allergic aspergillosis, where the pathological implications can be described as involving the absence of any contact between pathogen and extracellular matrix of the host, although damaged immune reactions result in *Aspergillus* species colonization in large and small airways. Therefore, the notion that ABPA is a true infectious disease remains contentious. Consequently, we wish to exclude ABPA from our discussion and focus on the pathophysiology of true infectious diseases caused by *Aspergillus* species. In the present review, we initially detail the current knowledge concerning IPA. We then describe CPA, which is considered a transitional form between IPA and SPA.

## 2. *Aspergillus* Species

*Aspergillus* species are classified into the fungal division Ascomycota. *Aspergillus* species are detected frequently in the ground, air, and in plants worldwide. Some species that exhibit virulence against humans include *Aspergillus fumigatus, Aspergillus niger, Aspergillus terreus*, and *Aspergillus flavus*. These species can be detected on the basis of characteristic conidial heads. However, in the case of IPA, conidial heads fail to be detected in histopathological specimens, while in the cases of CPA and SPA, conidial heads are seldom detected. Although the presence of calcium oxalate crystals resulting from *Aspergillus niger *has been detected, it has been known that it is not a specific finding of *Aspergillus niger*. However, it is exceptionally so rare to encounter the tissue including calcium oxalate that is caused by infection of *non-niger Aspergillus*. It remains controversial whether virulence differs between *Aspergillus fumigatus* and other species [7].

## 3. Aspergillosis

*Aspergillus* species are sometimes detected in the intrapulmonary region, auditory canal, nasal cavity, and cornea. In immunocompromised hosts, rare regions are involved following infection by *Aspergillus* species [8–16]. Two major subtypes are well known in the intrapulmonary region: IPA, which involves defense mechanisms of the host that are lowered by the innate course of the underlying disease and/or a requirement of induced immunosuppression, and SPA, which is usually caused by old tuberculosis or cystic disease of the lung. Another clinical entity has recently been proposed, namely, chronic pulmonary aspergillosis (CPA), which occurs when the defense mechanisms of the host are mildly impaired. However, various aspects of the background factors make the detection of this disease problematic, such as a paucity of clinical symptoms, indeterminate chest X-ray findings, and a low rate of isolation of fungi from sputum and other specimens obtained from the respiratory tract. Additionally, the general state of patients is often so poor that invasive laboratory tests are restricted, thus further limiting any concrete histopathological and cytological diagnosis. Given these circumstances, there is an urgent need to delineate the pathological mechanisms underlying pulmonary aspergillosis in order to establish effective diagnostic methods and facilitate the development of safer and more definitive therapeutic strategies.

## 4. Invasive Pulmonary Aspergillosis

The number of immunocompromised hosts has continued to increase in recent years due to a number of factors, including an increased occurrence of chemotherapy, HIV infection, organ transplantation, and long-term administration of immunosuppressants. The quick and correct diagnosis of immunocompromised hosts is particularly important [17–24]. In addition to adults, children may also be affected by IPA [25, 26]. Many reports have been published detailing the clinical and radiological features of IPA [27–32].

**Figure 1:**
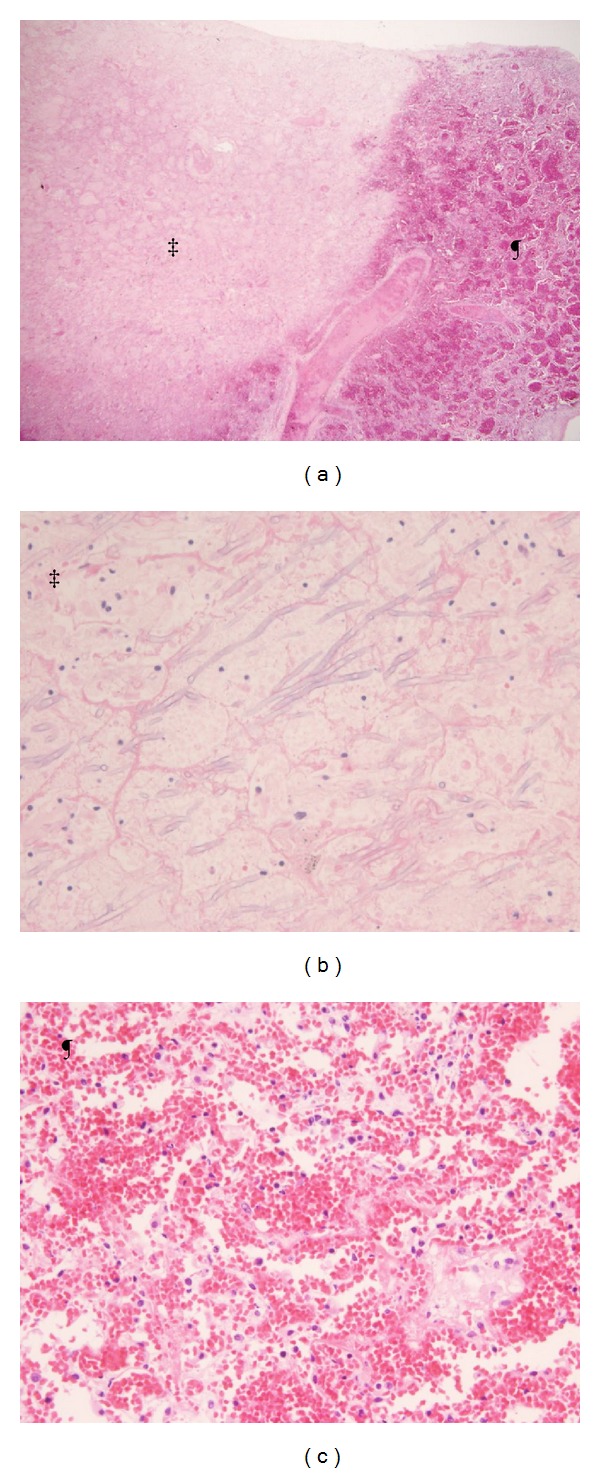
Invasive pulmonary aspergillosis, discrete nodule. (a) The nodule comprises coagulation necrosis of the lung tissue (‡) and acute hemorrhage usually accompanies the necrosis (¶) and may mimic a halo sign on CT (Hematoxylin-Eosin, X20). (b) Zone formation (mimicking an annual ring) of hyphae aligned in a radial pattern. Few inflammatory infiltrates are accompanied in the lesion (Hematoxylin-Eosin, X200). (c) Acute hemorrhage can be seen. No hyphae can be observed.

We have been conducting histopathological and pathophysiological investigations of IPA [33, 34]. Our results have led to the identification of two distinct patterns among the large group of patients investigated. One pattern involves a discrete nodule (DN) consisting of well-demarcated and round-shaped coagulation necrosis in which numerous hyphae are aligned in a radial pattern (Figure 1). A circumferential band of hemorrhage surrounds the area of coagulation necrosis. Less apparent is any type of inflammatory infiltrate in this pattern that usually occurs in a patient with severe bone marrow suppression or agranulocytosis.

**Figure 2:**
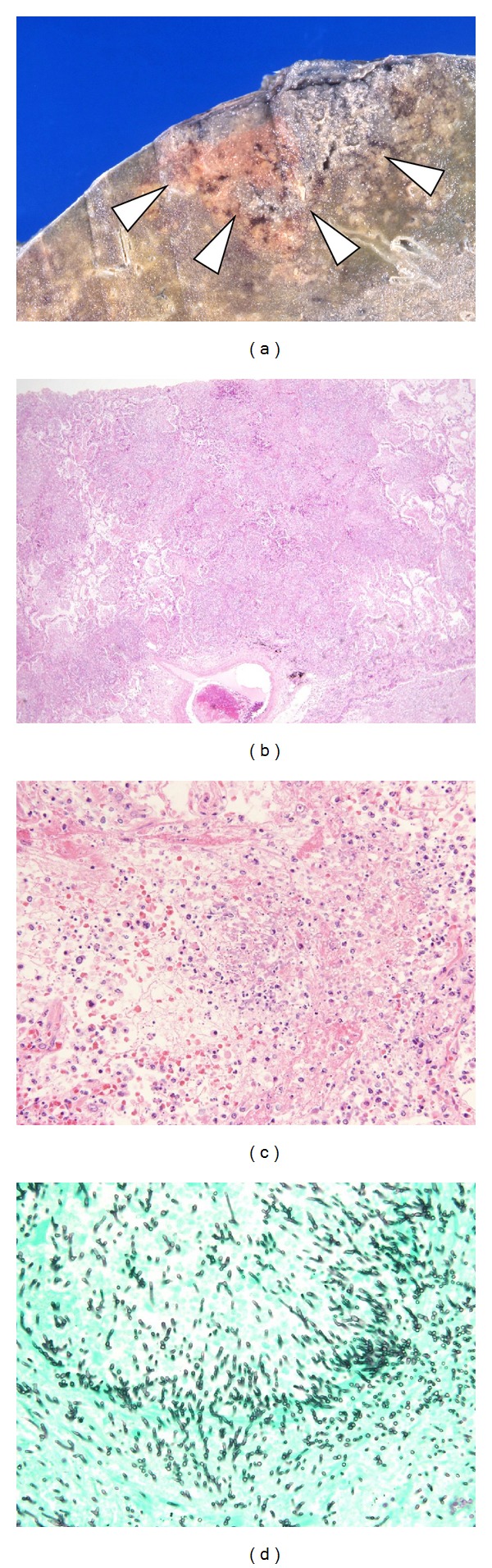
Invasive pulmonary aspergillosis, fused lobular consolidation. (a) The lesion comprising a fusion of solidified lobules is seen on a section of lung (white arrow head). The necrotic cavity is usually present at the center. (b) Ordinary bronchopneumonia can be seen (Hematoxylin-Eosin, X20). (c) Alveoli present due to the invasion of hyphae are filled with neutrophils (Hematoxylin-Eosin, X200). (d) Growth of fungus is present in the airspace (GMS, X400).

The second pattern involves fused lobular consolidation (FLC), which corresponds to the usual bronchopneumonia observed and is characterized histologically by the filling of acute inflammatory exudates with a fungal proliferation in alveoli (Figure 2). A gross feature of this pattern is fusion of lobular consolidation. Necrosis exhibited in FLC is usually colliquative and may be induced by neutrophilic infiltration. This can produce a cavity at the center of the region when the bronchi associated with the necrosis play a role in drainage. Patients indicating FLC maintain a considerable response to neutrophils, which is recognized as a first line of defense against *Aspergillus* species infection. 

**Figure 3:**
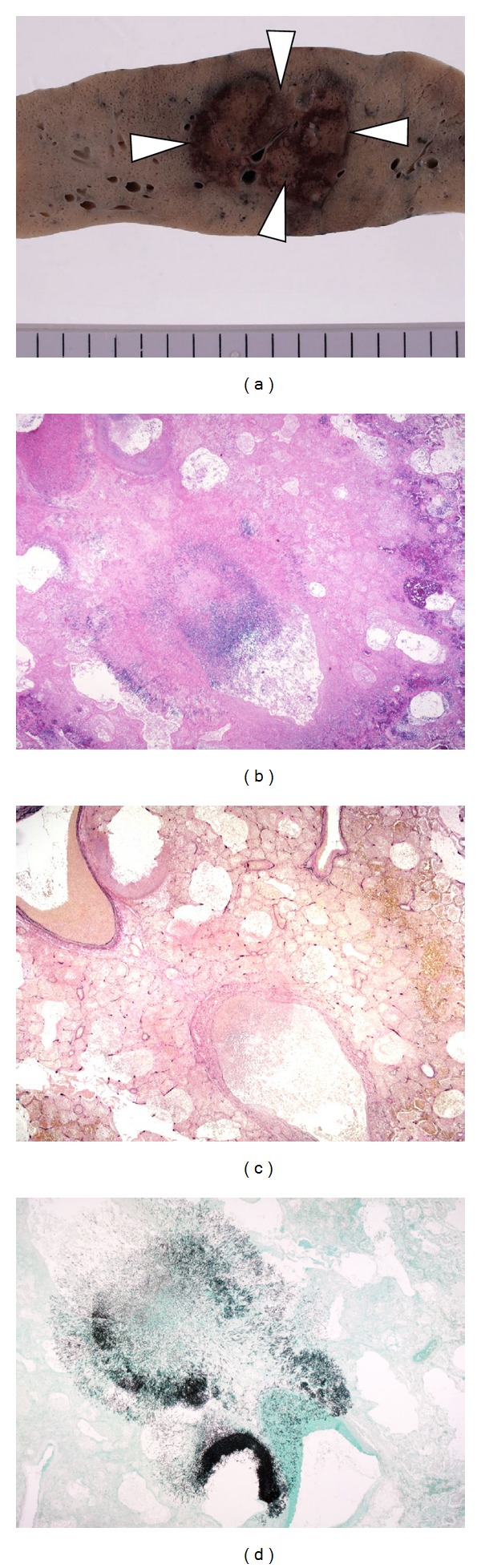
Invasive pulmonary aspergillosis with infarction. (a) There is a sharply demarcated nodule on a section of the lung (white arrow head). (b) Fungus hyphae can be seen in the vessel. Coagulation necrosis can be seen surrounding the vessel (Hematoxylin-Eosin, X20). (c) The pulmonary structure is not destroyed by the fungi (Elastica van Gieson, X20). (d) Hyphae aligned in a radial pattern within the vessel and airspace (GMS, X20).

**Figure 4:**
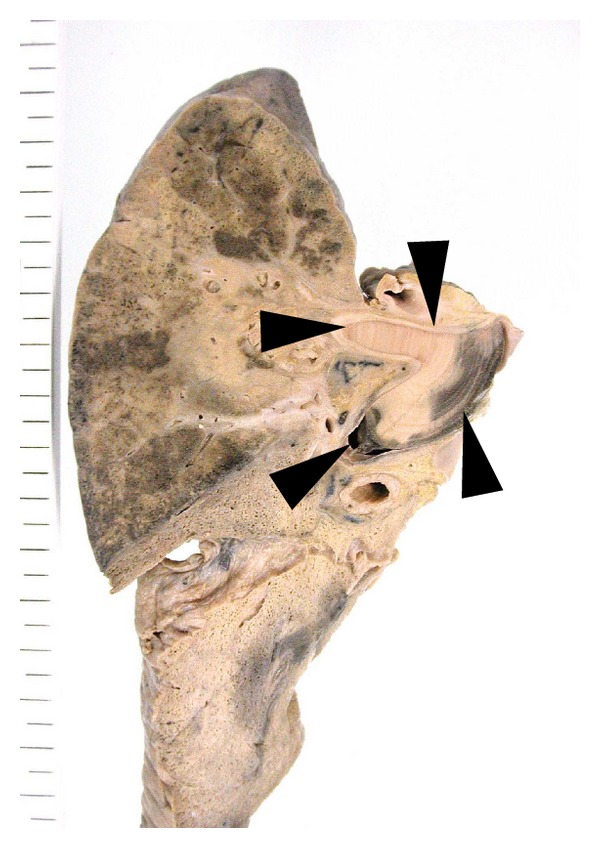
Invasive pulmonary aspergillosis with huge embolism. There is a huge embolism in the large vessel (black arrow head) with obstructive pneumonia. This was a case with long-term followup, and the annual ring can be seen in the embolism.

Using two radiographic images for each case, comprising initial alterations and the pattern just prior to death of the patient, the correlation between histopathological features and radiographic patterns was studied. Shadows documented as patchy/nodular or indicating irregular infiltrate were found as the commonest initial radiographic alteration in our patient group. Each abnormal shadow enlarged gradually or rapidly, and most of the patchy/nodular shadows transformed into irregular infiltrates. However, some of the closest parallel findings between initial radiographs and histopathological findings at autopsy included the discovery of a DN in patients who initially showed patchy/nodular shadows and the presence of FLC in patients who had irregular infiltrates as interpreted from radiographic images. Additionally, the halo sign is recognized as one important indicator of IPA and may mirror a band of hemorrhage surrounding a DN that developed in a patient with agranulocytosis. On the other hand, a cavity and peripheral air crescent may be caused by an exclusion of colliquative necrosis produced by neutrophilic infiltrate against the invading fungi. Additionally, some IPA cases indicate infarction (Figure 3) and huge embolism (Figure 4).

The halo sign is a special computed tomography (CT) finding consisting of a macronodule surrounded by a perimeter of ground-glass opacity [35, 36]. In immunocompromised patients at very high risk of invasive mold infection, the halo sign has long been considered an early sign of IPA. This characteristic is identified in a significant proportion of patients with IPA in whom the halo sign was a criterion for a probable diagnosis of IPA and is identified in about 60% of patients with IPA following initial CT imaging. The ground-glass component of the halo sign corresponds to hemorrhage surrounding the edge of the DN identified by histopathology. However, this sign is highly transitory. For example, in one longitudinal CT study of patients with IPA in whom 72% of patients showed a halo sign following initial CT analysis, only 22% of these patients still showed a detectable halo sign 10 days later. The CT halo sign is not unique to IPA. It has been reported in other angioinvasive mold infections, such as those involving species within the *Mucor, Trichosporon, Penicillium,* and *Fusarium*. The halo sign has also been reported in a wide variety of other infections and under noninfectious conditions. While there are a wide variety of conditions other than IPA that show a CT halo sign, it is important to recognize that IPA is by far the most common condition showing a CT halo sign in patients with severe impairment of the immune system. Some studies have indicated that treatment with preemptive anti-*Aspergillus* therapy following a CT halo sign finding can improve the outcome in patients with a compatible illness who are at high risk of invasive mold infection. IPA patients showing a CT halo sign have also exhibited better survival rates than those without a CT halo sign. Favorable responses to the treatment of patients in the halo-sign group have been found irrespective of the underlying condition category.

The characteristic transition lesion of IPA occurs after partial recovery of neutrophil function, by which time the DN has begun to develop liquefaction necrosis that is limited to its periphery. This process occurs because blood vessels around the margin of the DN remain patent. Thus, new neutrophils begin to be delivered to the nodule periphery, thereby facilitating liquefaction necrosis. The resulting cavitation separates the lung at the periphery of the persisting central zone of coagulation necrosis. The histopathology of this transition lesion forms the basis for the analogous radiological air crescent sign, which is an imaging indicator of late IPA. The air crescent sign is a specific type of cavitary lesion in which a semilunar pocket of gas surmounts a macronodule [37]. Since the appearance of this sign is generally known to coincide with recovery of neutrophil function, it is found as an initial CT finding in only a small proportion of patients with IPA. Like the CT halo sign, the air crescent sign is considered a specific indicator of IPA in patients at high risk of invasive mold infection. An investigation of patients with IPA revealed that the frequency of the air crescent sign increased slowly as the frequency of the halo sign rapidly diminished. 

It has recently been accepted that the appearance of a reversed halo sign (RHS) in CT images from immunocompromised patients is indicative of invasive fungal infection [38, 39]. RHS is characterized by a central ground-glass opacity surrounded by a rim of consolidation. We reported an invasive pulmonary mucormycosis case involving RHS, which showed coagulation necrosis of the alveolar septa and remaining air content in the central area with mirrored central ground-glass opacity. The periphery of the RHS lesion corresponding to the outer rim comprised a triplet structure: liquefaction, consolidation, and organization from the inner to outer layer. There were no elastic fibers in the liquefaction that developed at the junction area between coagulation necrosis and consolidation. The consolidation indicated that the second layer of the outer rim comprised mononuclear cells and multinucleated giant cells filling the alveolar space, although few polymorphonuclear leukocytes were observed. Although elastic fibers were confirmed in each septum in this layer, they were more or less altered. At the third layer of the outer rim, the alveolar space was replaced with collagen fibers, which represented a major skeleton of alveolar septa. There was no inflammatory infiltrate in this layer. In contrast, although the DN of IPA also exhibited central coagulation necrosis, the air content in the alveolar space was entirely replaced with exudation, which likely comprised plasma without leukocytes. The round-shaped coagulation necrosis was encompassed by an oozing of erythrocytes. 

The difference between the DN of IPA and RHS of invasive mucormycosis may be caused by the relationship between fungus proliferation and the vessel. In IPA cases, *Aspergillus* species grow straight towards the vessel. However, *Mucor* grows along the vessel in cases of mucormycosis. These differences in growth may influence the existence of an air remnant in the central area.

## 5. Simple Pulmonary Aspergilloma

**Figure 5:**
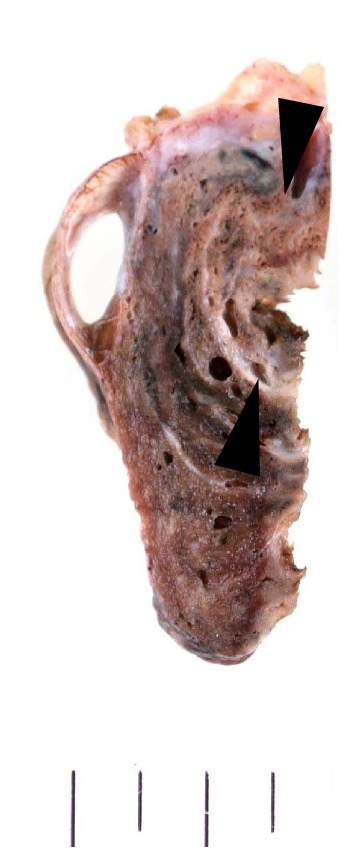
Simple pulmonary aspergilloma. There is a preexisting cavity (black arrow head) and few changes surrounding the cavity.

A noninvasive form of pulmonary aspergillosis known as SPA is defined pathologically as the development of a fungal ball in preexisting cavities usually caused by old tuberculosis or cystic diseases of the lung (Figure 5) [40]. Hyphae align in a compact fashion to form the radial pattern of the ball that develops in cavity possessing walls that are usually eroded or covered with metaplastic epithelium of the respiratory tract. The inflammatory infiltrate is essentially responsible for the chronic inflammation observed in the wall, and no hyphal invasion occurs when the patient is immunocompetent. On the basis of these observations, recognition of the entity is somewhat controversial since the histopathological definition of the disease requires the exclusion of invasive fungal proliferation into the lung tissue that must be an essential feature of the infectious disease. 

## 6. Chronic Pulmonary Aspergillosis 

**Figure 6:**
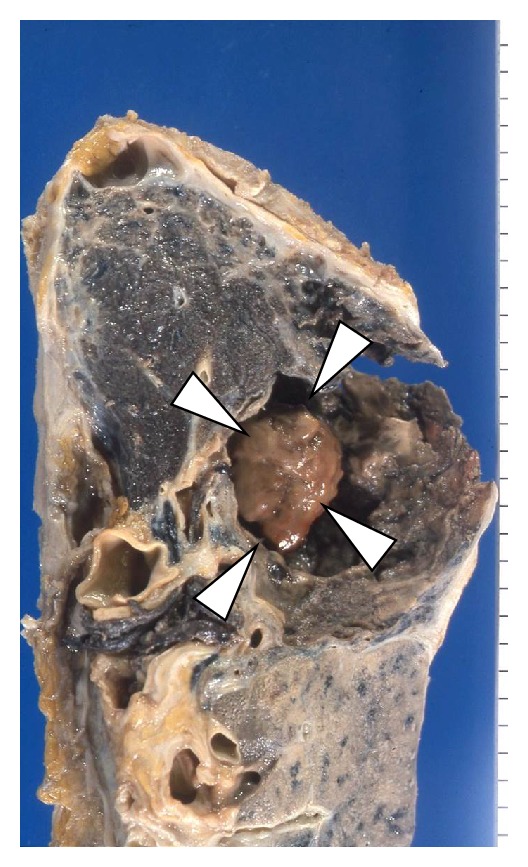
Chronic pulmonary aspergillosis. There is a cavity filled with fungus (white arrow head) surrounded by the secondary organization.

Although no general consensus has been reached regarding the nature of this form of aspergillosis, we wish to regard this form of aspergillosis as a state of *Aspergillus* infection with minimal invasion [41–44]. Elucidation of the semi-invasive form of aspergillosis remains limited. However, this form may be largely accepted as the state transformed from the noninvasive form into the invasive pulmonary disease when defense mechanisms of the host are lowered by the innate course of the underlying disease and/or a requirement of induced immunosuppression (Figure 6). The cavity wall is usually eroded and invaded by elongated hyphae. Acute and chronic inflammatory infiltrates are seen in association with fibrosis and necrosis in various degrees. Blood vessels are usually involved and are occluded by the invasion that may cause hemoptysis.

## 7. Conclusion

The present paper described the pathophysiology of aspergillosis. Since a large number of invasive *Aspergillus* species infections occur as opportunistic infections, there is a wide spectrum of histopathological features associated with lesions at the site of infection. Histopathology of the lesions can be understood as a phenotypical representation of the interaction between variously lowered defense mechanisms of the host and virulence of the invading fungi. Detailed observation with consideration of previous pathological knowledge regarding infection and inflammation can provide important information to predict the pathophysiology of the patient. Moreover, a consideration of the importance of the pathophysiology should be emphasized in an effort to assist with the implications of radiographic findings, clinical symptoms, and laboratory dates. By reviewing the studies reported, CT images in particular are found to accurately mirror the histological features of the lesion that can be recognized as a phenotypical representation of the pathophysiology of *Aspergillus* infection. This is also confirmed by reports emphasizing the importance of CT scans in identifying indicators of clinical signs and symptoms of the disease.
